# Food Processing at a Crossroad

**DOI:** 10.3389/fnut.2019.00085

**Published:** 2019-06-25

**Authors:** Dietrich Knorr, Heribert Watzke

**Affiliations:** ^1^Food Biotechnology and Food Process Engineering, Technical University Berlin, Berlin, Germany; ^2^Dr. Phil. Watzke Heribert Consulting, Lausanne, Switzerland

**Keywords:** food processing, ultra-processed foods, emerging technologies, PAN principles, food process-structure-function relationship, nutrient profiling, food system changes

## Abstract

Recently, processed foods received negative images among consumers and experts regarding food-health imbalance. This stresses the importance of the food processing—nutrition interface and its relevance within the diet-health debates. In this review, we approach the related questions in a 3-fold way. Pointing out the distinguished role food processing has played in the development of the human condition and during its 1.7 million year old history, we show the function of food processing for the general design principles of food products. Secondly, a detailed analysis of consumer related design principles and processing reveals questions remaining from the historical transformation from basic cooking into advanced food technology. As a consequence, we analyze new and emerging technologies in relation to their contributions to food-health impacts. During the last 35 years, new and emerging food technologies have initiated a paradigm shift away from conventional process methodologies to gentler, non-thermal processing. Reducing the existing uncertainties in the assessment of impact of technology like “minimal processing,” we propose the use of the newly established ISO standard for natural food ingredients as a “reference point.” Finally, we assess the usefulness of recently proposed classification systems, e.g., NOVA classification, based on comprehensive insights of recently published nutritional analysis of those classifications. This paper calls for a radical change and worldwide adaptation of the key research and developmental areas tackling the grand challenges in our food systems.

## Introduction

Food is on everyone's mind. At least, this is the impression given by the prominence food themes, products and consumer attitudes provided on social media. However, food available in their healthy-looking and picture-perfect presentations seem far off from the daily foods people get on their table. An excess of fads, myths, hypes, and food belief systems are of no help for consumers searching for clear, balanced and well-researched answers and advice.

While the impact of social media on actual food consumption is still uncertain (1), heated discussions can establish a dichotomy between what is thought to be natural-healthy foods and processed products, whereby the latter are stigmatized as unhealthy. In these views, processed foods acquired negative connotations and the interface between food processing and nutritional impact moved into center stage. Historically, food processing and nutrition have remained separate scientific endeavors. Questions of health-impact of processing create a need for a trans-disciplinary view and, as a consequence, a practical and simplified classification for consumers.

The need for simple classification of foods is a longstanding concern. The anthropologist Claude Levi-Strauss observed and demonstrated that the culinary triangle “raw-cooked-rotten” establishes a basic scheme not only for separating edible from non-consumable foods but for analysis of the larger world including social relations (2). The culinary triangle mirrors the transformations that food has been subjected to throughout human history. The reduction of “rotten” food was always on the mind of those handling food. Traditionally, food processing was developed for solving problems of long-time storage and transport of foods by various simple means (e.g., cooking, curing, smoking). Later, pasteurization and other heat treatment technologies achieved effective reduction of spoilage and pathogenic microorganisms rendering foods safe. Development of food processing in 20th century added processing for increasing palatability and production of indulgent products. Packaged foods inspired the search for and applications of new and intelligent packaging materials and the general question of shelf-life quality reached center stage of technology developments. The introduction of processing aids and food additives extended the scope of marketing oriented industrial food creations. Less attention was directed toward the food processing—nutrition interface that has only gained prominence in recent food-health debates.

In this review, we approach the questions arising from the food processing—nutrition interface in a 3-fold way. Firstly, the paper deals with the functions of food processing and their historical development and impact. Addressing the role and function of food processing for the general design principles of food products in detail allows the identification of short-comings and missing technology. From this analysis, it becomes clear that the health-related design principle lacks stronger support from food processing. This leads to the question as to how new and emerging technologies close the gaps identified and how they relate to requirements of nutritional qualities of products. Finally, we address the recent discussion of simplified classification systems, e.g., the NOVA classification, basing our analysis on recently published comprehensive nutritional reviews.

## The Role of Food Processing

The discussions about nutritional impact of food processing and its according classification underline the need for clarification on terminologies and processes, as well as on the need for information, stressing the role of processing in ensuring a safe, functional and nutritional food supply.

The need for clarification on processing gains more weight through the substantial market importance of food processing. Before the financial crisis in 2008, the assessment of the processed food market valued its size at US$ 3.2 trillion representing three-quarters of the worldwide food market. Global producing and trading of processed-packaged foods by global companies make up around 10% of that market, leaving 90% of processed food production to indigenous companies. This value amounts to ~8% of the total worldwide processed food production (3).

While the objective of processing of food is the provision of a safe and nutritional food supply, the ways to achieve this are manifold and have undergone rapid development in recent times (4). The function of food processing contributes to all stages of food making from raw material, ingredients and the production of the final product. It is not only confined to the last step from the formulation premix to end product (5). Moreover, it relates closely to cooking and gastronomic food manipulations as Aguilera pointed out in his comprehensive review (6). The versatility of food processing techniques and solutions also adapt to the varied needs of food producers worldwide.

### History of Food Processing

Throughout the human evolution, humans and their ancestors were confronted with difficulties to get to foods or to eat them in the raw state. As omnivorous species they lacked any specialization of a speedy predator nor did they efficiently digest plant materials. For this reason, humans have searched and with ingenuity they have developed means, tools and technologies to improve food availability, digestibility, safety, transportability, and storage life (7). Recent studies estimate the first use of fire between 2 million and 200,000 years ago. Fire and cooking allowed unlocking of caloric energy and access to nutrients (8, 9). Support for the thesis that changes in diets and the invention of cooking supported the growth of our brain to host 86 billion neurons comes from recent neuroscience studies (10). Starting with the invention of agriculture, food processing set off to a broad development of unit operations and processes embracing all edible food sources (4, 11). Technologies like refrigeration, pasteurization, sterilization and canning rank even under the most significant inventions in the history of food and drink in the UK Royal Society member and expert voting (12).

Already from the beginning, food processing was directed toward making foods safe, transportable, and retaining their dietary values trough storage. Fire and first preservation techniques (e.g., air drying, smoking, fermentation) transformed our species into a migrating species settling in any ecological niche on this planet. Population growth through improved agricultural yield, for example, crop rotation, demanded more extensive and more efficient preservation technology. McLachlan has proposed four major areas which food technology and its processing abilities were developing in McLachlan (13):

1. preservation (avoiding spoilage and foodborne diseases)
2. increase palatability (better tasting and access to nutrients)
3. transportation stability (development of supply chains)
4. production of convenience food (freeing from daily chores)

Thermal preservation technologies exhibit a fascinating history, beginning with drying and smoking, going back ~6,000 years. The industrial revolution saw pasteurization and sterilization (Pasteur and Appert) augmenting the scope and power of food preservations in the 19th century. Using cooling, ancient Romans, and Chinese applied ice blocks and snow to preserve delicate foodstuff (14). Stockfish, salmon and herring trades throughout medieval times depended on drying, smoking, but mostly on salting methods to make large traded quantities transportable (15).

### Food Processing Produces Specific Characteristics of Products

A non-negotiable basic principle guides food production: safety of food. The historical development of food technology is a key witness to solutions for this primordial prerequisite. However, from safe food production, particular products are produced in various forms and different kinds. They share a group of interdependent design principles that represent consumer preference areas. The abbreviation “C.H.E.F.S.” summarizes those principles. It stands for “convenience,” “health,” “epicurean/emotions,” “function,” and “sustainability” (16). These principles resonate with the consumer glossary descriptors, like “taste,” “natural,” and so on.

“Convenience” refers to the time economy modern food products provide to the consumer. The term “health” also includes, in its broadest sense, the basic and safe avoidance of any food-borne diseases beyond health claim driven functionality. The combination term “epicurean/emotions” relates to the sensorial qualities and the delightfulness of food. The term “function” collects food aspects designed to cater to specific eating occasions, including specific eating habits, for example at religious events. Finally, “sustainability” extends beyond the specific environmental impact of a given food product, including the organization of the complete food systems.

While the impact assessment of food processing on nutrition is already available, the inverse guiding information is missing. What do nutrition and healthy diets demand from food processing? If the goal of nutritional health targets “balanced dietary trajectories,” a new “culinary triangle” (minimal treated-processed-transformed) has to be supported by adapting food processing (16, 17). This point of view is supported by the fact that “food, not nutrients, is the fundamental unit in nutrition” as Jacobs and Tapsell have argued (18).

Knorr also recently stressed the requirements needed to gain consumer trust and acceptance (19). The processing group of the European Technology Platform: Food for Life (ETP 2007), a European food research vision document, introduced the PAN (reverse engineering) concept, suggesting that food processes must adapt to the preferences, acceptance, and needs of the consumers rather than adapting raw materials to the process requirements as previously applied (20).

McLachlan's list of food technology developments (13) can be matched up with the five design principles. The processing technologies support designs within the five domains. However, the comparison clearly shows that the technologies might have beneficial or adverse effects on a specific design. State-of-the-art product development tries to balance all design principles, keeping the trade-off as small as possible. Historically, the food industry has concentrated its efforts mainly in the design of highly palatable and convenient foods throughout the last 50 years.

### New Food Processes

Despite the long history of food processes, the above discussions and analysis make it clear that the future demands new food processes, unit operations, and production technologies. Fortunately, a large variety of novel and emergent unit operations are at disposal for the use in reduced and efficient processing of foods.

All processing-oriented food classification systems encompass processes described under the term “minimal processing.” Minimal processing is developed, especially on demand from restaurants, catering and foodservice industry, to provide pre-cut and pre-prepared vegetable and meat products for meal preparations, saving labor cost and improving hygiene (what is called “mise en place” in culinary art). However, pre-cut and pre-prepared consumer food products also appeared on the retail shelves (21).

A widely accepted definition is that minimal-processing technologies are modern techniques that provide sufficient shelf life to foods to allow their transport and distribution, while also meeting the consumer demands for convenience and fresh-like quality (22).

However, critical voices raised concerns against the industrial practice of minimal processing. For example, Stuckler and Nestle state that “although in theory, minimal processing of foods can improve nutritional content, in practice most processing is done to increase palatability, shelf-life, and transportability; processes that can reduce nutritional quality” (23).

Minimal processing has been the answer to the problem of keeping and, if possible, increasing the nutritional quality of foods. The term covers a large group of different technologies, all trying to reduce nutrient degradation during production and storage. The critical feature of minimal processing is the lower thermal load during unit operations to protect fragile food components (e.g., vitamins). However, calibrating the impact of a given technology on the preservation of nutritional quality is not an easy undertaking.

The newly published ISO standard “ISO/TS 19657” on natural food ingredients offers the needed “reference” (24). The technical criteria deciding the analysis of the naturalness of a food ingredient could determine a lower limit that could be more easily achieved. Technologies fulfilling the requirements of the standard could form a positive list of minimal processing unit operations by simultaneously achieving the required food safety standards. Furthermore, food profiling and descriptor methodologies could provide quantitative assessment of the loss of nutritional quality (25).

### New and Emergent Physical Technologies

The discussion so far has indicated that a reduced “processing load” can only be achieved by physical and biotechnological techniques. To accomplish the basis of any food product development producing safe food, a variety of physical technologies are available. Table 1 provides an overview of critical non-thermal technologies, including emerging ones that are distinguished by their non-thermal process nature. The table also contains the current status of development with descriptions of advantages and disadvantages in their application.

**Table 1:** Comparison of common non-thermal processes including emerging ones.

	**Fermentation**	**Irradiation**	**HP**	**PEF**	**A Plasma**	**US**	**UV**
Principles of action	Biotransformation (enzymes)	Oxidation free radicals	Activation volume	Transmembrane potential	Oxidation UV free radicales	Pressure, gradients, cavitation, sheer forces, free radicales	Ozone, free radical formation
Status	Industrial (~8 Ka)	Pilot/industrial	R&D industrial (~250 units)	R&D industrial (~40 units)	R&D industrial (medicine, BT)	Industrial	Industrial
Advantages	↑ digestion and edigility preservation	Low aw products	Quality, freshness 3rd dimension (p, T, t) Process opportunities	Quality, freshness, low energy. continuous	Universal (gas)	↑ mass transfer, partial microbial inactivation, ↑ extraction of components	Microbial inactivation
Disadvantages	Major product conversion	↑ free radicals ↓ consumer Acceptance no enzymes	Batch process	Aseptic packaging needed moisture required	Surface treatment	Energy intensive	Surface treatment cow penetration depth
Challenges	Mechanisms	Consumer acceptance	R&D continuous indicator microorganisms	R&D process integration indicator microorganisms	Proof of concept consumer acceptance indicator microorganism	Energy distribution temperature control	Oxidation reaction impact on sensory properties
Opportunities	New raw materials solid state	Low aw products	Composite materials small scale/home processing new fields (health), new raw materials	Scalable equipment new concepts new fields	Gas mictures new concepts gas (diffusivity)	Combination processes scalable equipment	Combination processes scalable equipment

*HP, hydrostatic pressure; PEF, pulsed electric fields; AP, atmospheric plasma; US, ultrasound; UV, ultra violet light*.

Their application provides gentle and efficient processing of raw materials, ingredients to complete products (26–29).

Böckel et al. have included in their review non-thermal processes from the broader scope of food processing and the beneficial aspects arising from them (30). A special place in the area of the novel and emergent technologies is taken by “pulsed electric fields treatment” technology (PEF), which targets its processing impact directly toward the biological weaknesses of cells. It perforates cell membranes and leads to high killing rates at lower temperatures (31–33).

The targeting of biological properties of raw materials permits a paradigm shift in food processing. The novel approach of “bio-guided processing” uses evolved intrinsic material properties, structures and dynamics to achieve the desired processing goal (34, 35). A typical example is the “extraction” of highly valuable PUFA oils from biomasses. Using edible oils instead of hexane allows for exchanging the PUFA oils against the carrier oil. The “extracting medium” becomes part of the final product. Vegetable oils have the same physicochemical solvability as hexane but exhibit lower solvability of biological molecules (e.g., phospholipids). A bio-guided extraction process results in a clear finalized oil product directly usable in-line production (34). The same concept transfers easily to other processing operations.

As mentioned in the European Vision document for food research (ETP 2007), two key drivers are at play for an adapted food technology (20). Firstly, food processing needs retargeting toward food structure—food functions/property development. This approach matches both adequate processing means with consumer needs expressed in C.H.E.F.S. principles. Examples of applying targeted technologies for creating a defined structure for providing tailor-made foods in areas, such as reduction of salt, sugar, and oil in foods, have been given by Janositz et al. (31), Raso et al (32), Kaufmann and Palzer (36), Hutchings et al. (17), and Fauster et al. (37).

The second paradigm changes, the PAN concept in its form of “reverse engineering,” is a radical shift from process/technology driven processing. This consumer-oriented approach, developed in the ETP “Food for Life” underwent further development by the newest edition of the European food research vision. It presents the current future-oriented strategy of the European Union. Other recent essential agenda points of European food science and technology include, transparency and integration of the food chain (20, 38), traceability and authenticity of our food supply (39), sustainability as an integral part of food processing (16, 19, 40, 41) and realization of the need for inter-disciplinary research (33).

## Classification of Food Processing

Recent consumer trends turn toward a stronger emphasis of the food-health balance of products. The myth and hypes of “super-foods” are only expressions of the consumer's concerns for healthier choices of products. Combining an existing food profiling system with processing related food descriptors permits the judgment of the trade-offs of food processing on the design of food products.

Recently, a new classification appeared in debates about the food-health imbalance based on stark terms like “ultra-processed foods” (42). The term coined by Monteiro classifies food products by the processing during their production (43–45). Monteiro and his collaborators declare those products as nutritionally empty and health-wise hazardous. The classification using this term should allow consumers to select healthy products that use a decreased intensity of processing during their production (43, 46, 47).

The newly created and currently frequently used term “ultra-processed foods” is receiving attention from consumer organizations and various media. The proponents of this new term use it to point to the processing of foods as one of the causes for non-communicable diseases (e.g., obesity, diabetes type 2, cancer, etc.) (48, 49). This relation underlines the need for clarification on terminologies and processes as well as the need for information, stressing the role of processing in ensuring a safe, functional, and nutritional food supply.

### NOVA Classification

Monteiro and collaborators developed a novel classification system from the distinction between processed and unprocessed food. Monteiro justifies the guidelines for healthier food choices with his observation that “…almost all work on nutrition and public health has overestimated the significance of nutrients and foods as such, and has underestimated or even overlooked the significance of processing…” (42).

The NOVA classification comprises four classes of foods of increasing processing intensities. The “ultra-processed foods,” UPFD, are defined as follows: “Ultra-processed foods were defined as industrial formulations, which, besides salt, sugar, oils, and fats, include substances not used in culinary preparations, in particular additives used to imitate sensorial qualities of minimally processed foods and their culinary preparations.” The presence of salt identifies ultra-processed foods, but also added sugar, high-fat content, additives, taste compounds, colors, substances from contact with packaging materials, and compounds produced during processing and storage (44).

The definition does not refer to processing or unit operations used during production of the described food products at all, but still remains in the realm of nutritional compositions and puts the emphasis on formulation and additives. Importantly, the consumer must be able to extrapolate the diminished nutritional quality from the presence of an additive or in-process (or storage) generated compound in a product (50). Only then does it count for the class of ultra-processed foods.

It becomes clear from the examples in NOVA that the distinction between unprocessed or minimal processed food products and ultra-processed foods boils down to whether they are produced through home cooking/culinary art or on an industrial scale. The NOVA classification builds on the idea that extended amounts of sugar, salt and fat, but also the use of additives, is the critical distinction against industrial processing of foods but not in any other form of food making. Table 2 summarizes examples of foods falling in the different classes of unprocessed or minimally processed foods and ultra-processed foods (44).

**Table 2:** Food products as stand-in examples for selected industrial processing [after (44)].

**Food group**	**Example**
Group 1: Unprocessed or minimally processed foods	Fresh, chilled, frozen, vacuum-packed fruits, vegetables, fungi, roots and tubers; grains (cereals) in general; fresh, frozen and dried beans and other pulses (legumes); dried fruits and 100% unsweetened fruit juices; unsalted nuts and seeds; fresh, dried, chilled, frozen meats, poultry and fish; fresh and pasteurized milk, fermented milk such as plain yogurt; eggs; teas, coffee, herb infusions, tap water, bottled spring water
Group 3: Ultra-processed food products	Breads, biscuits (cookies), cakes and pastries; ice cream; jams (preserves); fruits canned in syrup; chocolates, confectionery (candies), cereal bars, breakfast cereals with added sugar; chips, crisps; sauces; savory and sweet snack products; cheeses; sugared fruit and milk drinks and sugared and “no-cal” cola, and other soft drinks; frozen pasta and pizza dishes; pre-prepared meat, poultry, fish, vegetable and other “recipe” dishes; processed meat including chicken nuggets, hot dogs, sausages, burgers, fish sticks; canned or dehydrated soups, stews and pot noodle, salted, pickled, smoked or cured meat and fish; vegetables bottled or canned in brine, fish canned in oil; infant formulas, follow-on milks, baby food

Martinez et al. state “NOVA is the food classification that categorizes foods according to the extent and purpose of processing, rather than in terms of nutrients” (50). This statement is most confusing to any food scientist because the applied categorization is not based on the extent or purpose of processing.

More recently, Martinez et al. suggested that food processing as such is not the issue (50). However, they indicated that a combination of unprocessed or minimally processed foods and processed ingredients result in “ultra” processed foods (44). In consequence, this would mean that the use of additives (including added sugar and salt), rather than the right food processing of agricultural raw material, determines the characteristics of ultra-processed foods. This definition would strangely and instantly transform a minimally processed yogurt into an ultra-processed product when the consumer sweetens it with additional sugar.

### Nutritional Analysis of NOVA Classification

The NOVA classification should function as a replacement for nutritional and dietary guidelines with the goal of aiding the consumers in making healthier food choices. An appraisal of this new classification system has to start with a comparison against other existing food classification systems based on food processing. Gibney et al. have undertaken a comprehensive and critical appraisal of the Nova classification system (51).

Gibney et al. remark that the definition of ultra-processed foods (see above) poses definition problems because the term “formulation” is open to various interpretations. Moreover, there is no mention of cut-offs per gram or portion or energy of defining food components of salt, sugar, fat, and additives. Furthermore, Gibney et al. point out that “…neither the terms used to define UPFDs nor the list of typical foods in each category of the NOVA system meet the normal standards set in established food classification” (51). For example, the European Food Authorities have developed a food classification standard (Foodex) “…to define foods in a way that suits all users of food-intake data from food chemical exposure to food intake for dietary purposes…” (52).

A further food classification system exists, which emphasize food processing in their classification schemes. The EPIC (European Prospective Investigation into Cancer and Nutrition) consortium based their direct coding of individual foods and meal components on the degree of food processing (53). The EPIC consortium applied their classification across a wide variety of cultures and gastronomic traditions. Moreover, the EPIC system allows the direct comparison with NOVA because both methods served in cancer-related studies (53, 54). A highly sophisticated food classification system is available in the food coding system “LanguaL” from EuroFIR. It comprises a large number of descriptors for different aspects of food processing (25, 53, 55, 56). The availability of broad and versatile food coding systems oriented to processing reveals the simplistic and contrasting approach of the NOVA classification scheme.

Gibney et al. concluded that the proposed NOVA classification system, which is pushed by its proponents for use in the UN Sustainable Development Goals and the UN Decade of Nutrition, is built on the irresponsible myth that the modern approach to food classification is static and out of date. Nova classification does not substitute epidemiological studies for ingredient impacts on public health-related issues. Finally, Gibney et al. point out that no data exist proving average consumer's ability “…in terms of income, culinary skills, available culinary facilities, and time or food availability…” to uphold the case that the abandonment of ultra-processed foods would significantly change nutritional well-being (51).

Various combinations between food profiling systems and food descriptor databases, like LangaL, are available. The thesaurus of LangaL comprises a very detailed list of food descriptors for processing terms. For example, the main term “preservation methods” enlists 81 sub-terms structured in a hierarchical organization up to 6 layers deep. LangaL provides a high resolution in its description of food items to which compositional and dietary quality data can be associated for guideline purposes (18, 25, 38, 57–59).

As Gibney et al. have already concluded in their appraisal, the existing systems are detailed enough to allow the assessments of the nutritional and compositional impact of food processing on diets (51). The NOVA classification system appears crude in comparison and its superficiality is misleading.

Miller Jones comes to similar and confirming conclusions in her recent article. She points to the tautological definition of ultra-processed foods and warns that significant micronutrient deficiencies will result in the avoidance of specific ultra-processed foods (e.g., whole-grain enriched bread) (60).

## Conclusions and Outlook

In response to consumer trends for healthier food choices, a close assessment of the impact of food processing is necessary. The existing food profiling systems and the food descriptors allow a very detailed identification of process treatments of ingredients, products, and meals.

However, terms like “ultra-processed food” are more misleading than explanatory. The proponents of the NOVA classifications system overlook the fact that a strict interpretation of their classifications scheme leads to the statement that the human body reacts to processing and not to nutrients, including those which appear during processing (50).

Consumers also bear part of the responsibility for choosing healthier foods, a fact very often forgotten in on-going discussions. Studies in behavioral economy, especially those of Richard Thaler, Nobel laureate in economics 2017, have shown that a slight push, a so-called “nudge,” toward a specific choice increases the chance of its realization. A typical example along these lines is the work of Walmsley et al. who could show that repositioning the vegetable stall closer to the entrance of a grocery store increases the vegetable sales by 15% (61). As they report, changing the “choice architecture” has a significant influence on the choice's consumers make.

To move forward in food research and development, a worldwide adaptation of the critical research and the development of agendas are required. A forceful paradigm shift is needed in the field of food process science and technology, to tackle the grand challenges stemming from the interface food processing with nutrition (19, 62). Most importantly, a paradigm change is necessary for food research. The specialization is detrimentally opposing progress in addressing the unsolved questions in food science and nutrition (63).

## Author Contributions

DK and HW developed concept, content and write-up of the article.

### Conflict of Interest Statement

The authors declare no conflict of interest. HW is owner of the Dr. Phil. Watzke Heribert consulting company.

## References

[1] Adglow The Impact on Social Media on Food Industry. (2018) Available online at: https://www.adglow.com/blog/the-impact-of-social-media-in-the-food-industry

[2] Levi-Strauss C The culinary triangle. In: Counihan C Van Esterik P editors. Food and Culture, A Reader. 3rd edn New York, NY; London: Routledge (2013). p. 40.

[3] Regmi A Gehlhar MJ New Directions in Global Food Markets. Washington, DC:USDA (2005)

[4] Brennan JG Butters JR Cowell ND Lilly AEV Food Engineering Operations. London (1969).

[5] Alexander E Yach D Mensah GA. Major multinational food and beverage companies and informal sector contributions to global food consumption: implications for nutrition policy. Global Health. (2011) 7:26. 10.1186/1744-8603-7-26 21806827 PMC3160359

[6] Aguilera JM Relating food engineering to cooking and gastronomy. Compr Rev Food Sci Food Safety. (2018) 2018:12361 10.1111/1541-4337.12361 33350113

[7] Ludwig DS. Technology, diet, and the burden of chronic disease. JAMA. (2011) 305:1352–3. 10.1001/jama.2011.380 21467290

[8] Wrangham R Catching Fire. How Cooking Made us Human. London: Profile Books Ltd (2009)

[9] Brown KS Marean CW Herries AIR Jacobs Z Tribolo C Braun D. Fire as an engineering tool of early modern humans. Science. (2009) 325:859–62. 10.1126/science.1175028 19679810

[10] Herculano-Houzel S The Human Advantage–A New Understanding of How Our Brain Became Remarkable. Cambridge, MA; London: MIT Press (2016).

[11] Kipple KF Ornelas KC editors. The Cambridge World History of Food, Vol 2 Cambridge: AdglowCambridge University Press (2000).

[12] The Royal Society. (2012). Available online at: https://royalsociety.org/news/2012/top-20-food-innovations/

[13] McLachlan T. History of food processing. Progr Food Nutr Sci. (1975) 1:461–91. 772753

[14] Knorr D Sinskey A. Biotechnology in food production and processing. Science. (1985) 229:1224–9. 10.1126/science.229.4719.1224 17770800

[15] Hoffmann RC A brief history of aquatic resource use in medieval Europe. Helgol Mar Res. (2005) 59:22–30. 10.1007/s10152-004-0203-5

[16] Watzke H New maps for healthy dietary trajectories and food product innovations, Sight Life Magazine. (2018) 32:78–83. Available online at: https://sightandlife.org/wp-content/uploads/2018/08SightandLifeMagazine_ProductInnovation_2018_NewMapsforHealthyDietaryTrajectoriesandProduct-Innovation.pdf (accessed June 12, 2019).

[17] Hutchings SG Low JYQ Keast RSJ Sugar reduction without compromising sensory perception. An impossible dream? Crt Rev Food Sci Nutr. (2018) 21:1–21. 10.1080/10408398.2018.1450214 29561168

[18] Jacobs DR Tapsell LC Food, not nutrients, is the fundamental unit in nutrition. Nutr Rev. (2007) 65:439–50. 10.1111/j.1753-4887.2007.tb00269.x 17972438

[19] Knorr D Khoo CSH Augustin MA. Food for an urban planet: challenges and research opportunities. Front Nutr. (2018) 4:73. 10.3389/fnut.2017.00073 29404333 PMC5780399

[20] ETP European Technology Platform Food for Life. Strategic Research Agenda 2007–2020. Brussels, BE: CIAA (2007)

[21] Ahvenainen R Hurme E Minimal processing of vegetables. In: Ahvenainen R Mattila-Sandholm Ohlsson T editors. Minimal Minimal Processing of Foods, VTT Symposium 142, VTT Biotechnology and Food Research Finland Kirkkonimmi: Finland (1994). p. 17–35.

[22] Ohlsson T Minimal processing-preservation methods of the future: an overview. Trends Food Sci Techn. (1994) 5:341–4. 10.1016/0924-2244(94)90210-0

[23] Stuckler D Nestle M. Big food, food systems, and global health. PLoS Med. (2014) 9:e1001242. 10.1371/journal.pmed.1001242 22723746 PMC3378592

[24] Definition and Technical Criteria for Food Ingredients to be Considered as Natural. International Standard Organization (2017).

[25] Ireland JD Moller A. LanguaL food description: a learning process. Eur J Clin Nutr. (2010) 64(Suppl. 3): S44–8. 10.1038/ejcn.2010.209 21045849

[26] Hendrickx MEG Knorr D Ultra-High Pressure Treatment of Foods. New York, NY: Klyver Academic; Plenum Publishers (2002). 10.1007/978-1-4615-0723-9

[27] Jermann C Koutchma T Margas E Leadley G Ros-Polski V Mapping trends in novel and emerging food processing technologies around the world. Innov Food Sci Emerg Technol. (2015) 31:14–27. 10.1016/j.ifset.2015.06.007

[28] Yaldagard M Mortazavi SA Tabatabale F The principles of ultrahigh-pressure technology and its application in food processing (preservation: a review of microbial and quality aspects). Afr J Biotechnol. (2008) 7:2739–67.

[29] Awad TS Moharram HA Shaltout OE Asker D Youssef MM Applications of ultrasound in analysis, processing and quality control of food: a review, Food Research International (2012) 48:410–27. 10.1016/j.foodres.2012.05.004

[30] Van Boeckel M Fogliano V Pellegrini N Stanton C Scholz G Lallje S A review on the beneficial aspects of food processing. Mol Nutr Food Res. (2010) 54:1215–47. 10.1002/mnfr.200900608 20725924

[31] Janositz A Noack AK Knorr D Pulsed electric fields and their impact on the diffusion characteristics of potato slices. LWT Food Sci Technol. (2011) 44:939–49. 10.1016/j.lwt.2011.04.006

[32] Raso J Frey W Pataro G Knorr D Teissie J Miclavcic D Recommendations and guidelines on key information to be reported in studies of application of PEF technology in food and biotechnology processes. Innovat Food Sci Emerging Technol. (2016) 37:312–21. 10.1016/j.ifset.2016.08.003

[33] Jäger H Knorr D Pulsed electric fields treatment in food technology: Challenges and opportunities. In: Miklavcic D editor. Handbook of Electroporation. Cham: Springer (2017) 2657–2680.

[34] Ward RE Watzke HJ Jiminez-Flores R German JB Bioguided processing: a paradigm change in food production. Food Technol. (2004) 58:44–8.

[35] Wang J Bertholet J Bruce German J Watzke H Soft processing for high-value edible oils. Lipid Technol. (2004) 16:225–8.

[36] Kaufmann SFM Palzer S Food structure engineering for nutrition, health, and wellness. Procedia Food Sci. (2011) 1:1479–86. 10.1016/j.profoo.2011.09.219

[37] Fauster T Schlossnikl D Rath F Ostermeier R Teufel F Toepfl S Impact of pulsed electric field (PEF) pretreatment on process performance of industrial French fries production. J Food Eng. (2018) 235:16–22. 10.1016/j.jfoodeng.2018.04.023

[38] Schiefer G Deiters J Transparency for Sustainability in the Food Chain. Bonn:University Bonn (2013).

[39] Yiannas F A new era of food transparency powered by blockchain. Innovations. (2018)12:46–56. 10.1162/inov_a_00266

[40] Kaput J Kussmann M Mendoza Y Le Coutre R Cooper K Roulin A Enabling nutrient security and sustainability through system research. Gene Res. (2015) 10:12 10.1007/s12263-015-0462-6 PMC4398674 25876838

[41] Espinoza-Orias N Roulin A Watzke H Cooper K Connecting the dots: assessing sustainable nutrition at Nestlé. In: Schenck R Huizen D editors. Proceedings of the 9th International Conference on Life Cycle Assessment in Agri-Food Sector (LCA Food 2014). San Francisco, CA; Vashon, WA: ACLA (2014). p. 380–9.

[42] Monteiro CA Nutrition and health. The issue is not food, nor nutrients, so much as processing. Public Health Nutr. (2009) 12:729–31. 10.1017/S1368980009005291 19366466

[43] Monteiro CA Cannon G Levy RB Claro RM Moubarac JC Ultra-processing and a new classification of foods. In: Neff R editor. Introduction to US Food System. Public Health, Environment, and Equity. San Francisco, CA: Jossey Bass A Wiley Brand (2015). p. 338–9.

[44] Monteiro CA Levy RB Moreira Claro R de Castro IRR Cannon G. A new classification of foods based on the extent and purpose of their processing. Cade Saude Publ. (2010) 26:2039–49. 10.1590/S0102-311X2010001100005 21180977

[45] Monteiro CA Cannon G Levy RB Claro R Moubarac JC Martins AP. The food system. Processing the big issue for disease, good health, well-being. World Nutr. (2012) 3:527–69.

[46] Monteiro CA We should eat freshly cooked meals. BMJ. (2018) 361: k2463 10.1136/bmj.k3099 30006451

[47] Monteiro CA Maubarac JC Levy RB Canella DS da Costa Louzada ML Cannon G. Household availability of ultra-processed foods and obesity in nineteen European countries. Public Health Nutr. (2018) 21:18–26. 10.1017/S1368980017001379 28714422 PMC10260838

[48] Monteiro CA Moubarac J-C Cannon G Ng SW Popkin B. Ultra-processed products are becoming dominant in the global food system. Obes Rev. (2013) 14:12107. 10.1111/obr.12107 24102801

[49] Moubarac JC Parra DC Cannon G Monteiro CA. Food classification systems based on food processing: significance and implications for policies and actions: a systematic literature review and assessment. Curr Obes Rep. (2014) 3:256–72. 10.1007/s13679-014-0092-0 26626606

[50] Martinez Steele E Baraldi LG da Costa Louzada ML Moubarac J-C Mozaffarian D Monteiro CA. Ultra-processed foods and added sugars in the US diet: evidence from a nationally representative cross-sectional study. BMJ Open. (2016) 6:e009892. 10.1136/bmjopen-2015-009892 26962035 PMC4785287

[51] Gibney M Forde CG Mullally D Gibney ER. Ultra-processed foods in human health: a critical appraisal. AmJ clinNutr. (2017) 106:717–24. 10.3945/ajcn.117.160440 28793996

[52] European Food Safety Authority The Food Classification and Description System FoodEx2. Revision 2. Parma: EFSA Supporting Publication (2015).

[53] Slimani N Deharveng G Southgate DA Biessy C Chajes V van Bakel MM. Contribution of highly industrially processed foods to the nutrient intakes and patterns of middle-aged populations in the European Prospective Investigation into Cancer and Nutrition study. Eur J Clin Nutr. (2009) 63 (Suppl. 4): S206–25. 10.1038/ejcn.2009.82 19888275

[54] Fiolet T Srour B Sellem L Kesse-Guyot E Allès B Méjean C. Consumption of ultra-processed foods and cancer risk: results from NutriNet-Sante prospective cohort. BMJ. (2018) 360:k322j. 10.1136/bmj.k322 29444771 PMC5811844

[55] Finclas P Ireland J Møller A Harmonised Food Descriptors - Eurofir Experience to Date. (2018). Available online at: https://www.efsa.europa.eu/sites/default/files/event/documentset/fc100623p2.pdf

[56] Becker W Møller A Ireland J Roe M Unwin I Pakkala H Proposal for Structure and Detail of a EuroFIR Standard on Food Composition Data. II: Technical Annex. Danish Food Information (2008).

[57] LangaL EuroFIR websites. (2018). Available online at: http://www.langual.org and http://www.eurofir.org/food-information/

[58] Darmon N Vieux F Maillot M Volatier JL Martin A. Nutrient profiles discriminate foods according to their contribution to nutritionally adequate diets: a validation study using linear programming and the SAIN, LIM system. J Clin Nutr. (2009) 89:1227–36. 10.3945/ajcn.2008.26465 19211815

[59] Vlassopoulos A Masset G Charles VR Hoover C Chesneau-Guillemont C Leroy F. A nutrient profiling system for the (re)formulation of global food and beverage portfolio. Eur J Nutr. (2017) 56:1105–22. 10.1007/s00394-016-1161-9 26879847 PMC5346408

[60] Miller Jones J Food processing: criteria for dietary guidance and public health? Proc Nutr Soc. (2018) 78:4–18. 10.1017/S0029665118002513 30249309

[61] Walmsley R Jenkinson D Saunders I Howard T Oyebode O Choice architecture modifies fruit and vegetable purchasing in a university campus grocery store: time series modeling of a natural experiment. BMC Public Health. (2018) 18:1 10.1186/s12889-018-6063-8 PMC6167822 30285680

[62] Khoo CS Knorr D. Grand challenges in nutrition and food science technology. Front. Nutr. (2014) 1:4. 10.3389/fnut.2014.00004 25988108 PMC4429640

[63] Knorr D Khoo CS. Food science without borders. Front Nutr. (2015) 2:33. 10.3389/fnut.2015.00033 26557645 PMC4614281

